# "In Vivo" Effects of Lithium Sulphate on the Turnover of Rat Tissue Nucleotides

**DOI:** 10.1155/MBD.1996.123

**Published:** 1996

**Authors:** Felicia Loghin, Carmen Socaciu, Sorin E. Leucuta

**Affiliations:** 1 Faculty of Pharmacy University of Medicine and Pharmacy “luliu Hatieganu” Cluj-Napoca Romania; 2 Department of Biochemistry University of Agricultural Sciences and Veterinary Medicine, str.Mănăştur 3 Cluj-Napoca 3400 Romania

## Abstract

The effects of lithium sulphate following acute administration to Wistar rats - in therapeutic and toxic doses - were investigated, using as markers the levels and turnover of brain, kidney and liver nucleotides. Adenine- and guanine-dependent nucleotides were assayed concomitantly, using a high- performance liquid chromatographic method. Following acute administration, lithium's effects on 3’,5’- cAMP levels were more significant at hepatic and cerebral level, in a dose-dependant manner. The effect on 3’,5’-cGMP had tissue specificity and was not dose-dependant. It might be possible that 3’,5’-cGMP is a better indicator of lithium toxicity than 3’,5’-cAMP.


### "IN VIVO" EFFECTS OF LITHIUM SULPHATE ON THE TURNOVER OF RAT TISSUE NUCLEOTIDES

Felicia Loghin[1], Carmen Socaciu*[2] and Sorin E. Leucuta[1]

[1] Faculty of Pharmacy, University of Medicine and Pharmacy "Iuliu Hatieganu", Cluj-Napoca
[2] Department of Biochemistry, University of Agricultural Sciences and Veterinary Medicine, str.Mănăştur 3, 3400 Cluj-Napoca, Romania

### ABSTRACT

The effects of lithium sulphate following acute administration to Wistar rats - in therapeutic and toxic doses - were investigated, using as markers the levels and turnover of brain, kidney and liver nucleotides. Adenine- and guanine-dependent nucleotides were assayed concomitantly, using a high-performance liquid chromatographic method. Following acute administration, lithium's effects on 3',5'-cAMP levels were more significant at hepatic and cerebral level, in a dose-dependant manner. The effect on 3',5'-cGMP had tissue specificity and was not dose-dependant. It might be possible that 3',5'-cGMP is a better indicator of lithium toxicity than 3',5'-cAMP.

### INTRODUCTION

Lithium is used as a major drug in psychiatry, especially in the treatment and prophylaxy of bipolar affective disorder, also in antidepressant-resistant patients, as well as in internal medicine[1-4].Despite almost 40 years of clinical use and scientific investigation, neither the pharmacological actions of lithium at therapeutic doses, nor its mechanisms of toxicity have yet been completely established.

According to the most recent theories, the pharmacological and some of the toxic effects of lithium are related to its interaction with the signal transduction pathways[5,6]. $Li^+$ has been shown to interfere both with G-proteins and with the turnover of second messengers like 3',5'-cAMP, 3',5'-cGMP and the molecules derived from phosphatidylinositol 4,5-bisphosphate ($PIP_2$) pathway[7-10].

Generally, lithium is known to inhibit the adenylate cyclase (AC) which catalyses the formation of 3',5'-cAMP, a second messenger. The differential inhibition of receptor-specific AC species could provide an unifying hypothesis for the therapeutic and adverse effects of the drug[5]. Thus, the inhibition of adrenoceptor-activated cyclase at low lithium concentration could be seen as therapeutic effect, while the inhibition of other systems, e.g. dopamine-stimulated, ADH-stimulated and TSH-stimulated cyclase

Lithium effect on 3',5'-cGMP was studied only in the brain and at therapeutic doses, without correlating the results with the effects on other cyclic nucleotides[5,11].

Considering the role of the second messengers in the developement of toxic phenomena, we investigated the effects of lithium sulphate following acute administration to Wistar rats, at therapeutic and toxic doses, using as markers the level and turnover of brain, kidney and liver nucleotides.

### MATERIALS AND METHODS

*Animals.* Four groups of male Wistar rats (body weight 240±25g) (provided by Animal Breeding Station, UMF Cluj-Napoca) were used, standard food and microclimate conditions being provided before and during the experiments.

*Treatment.* Lithium sulphate was dissolved in distilled water and given orally by intubation. Three doses [ 2 (group B), 6 (group C) and 10 (group D) mmol $Li^+$/kg body weight ] were applied by single administration to experimental groups. Controls (group A) received distilled water.

*Sampling.* All the animals were sacrified 48 hours after the treatment and immediately the whole brain, kidney and liver were taken on ice for nucleotide assay.

*Extraction of nucleotides* from all tissues was performed according to Perret method[12], by protein precipitation with trichloroacetic acid 10%, centrifugation and neutralization of supernatant with potassium hydroxide 1M. The lipids were removed by extraction with diethylether, before acid precipitation.

*Nucleotide separation* was performed using a combination of two HPLC methods[13,14]. The separation was performed with a gradient HPLC system, using two Altex 101 pumps, an ODS column (200 x 4 mm i.d.and 5 μm particle diameter), a UV-VIS Kontron Instruments detector, coupled to a Hewlett Packard 3650 integrator. All chromatograms were registered at 254 nm. The flow rate of mobile phases was 1,5 ml/min and the elution of the samples was performed with a step-gradient of 0.05 M $NH_4H_2PO_4$ (solvent A, pH=6) (0 - 7.5 min) and a mixture 90:10 of 0.05 M $NH_4H_2PO_4$ : Methanol (Solvent B, pH=5.5) ( 7.5 - 14 min).

*Reagents.* Reagent-grade nucleotides (GTP, GDP, ATP, ADP, 5'-AMP, 3'-AMP, 3',5'-cAMP, 3',5'-cGMP) and $NH_4H_2PO_4$ were purchased from Sigma and methanol of HPLC purity from Merck.

### RESULTS AND DISCUSSION

The identification of the nucleotides in the biological samples was done according to their retention time ($t_R$) and the order of elution comparared with standards' separation (Fig.1). Co-chromatograms of each sample with different standard nucleotides were also performed for a better identification of peaks.

For the quantitative analysis, the chromatographic peaks were integrated and from their area, the percentage of each nucleotide was determined.

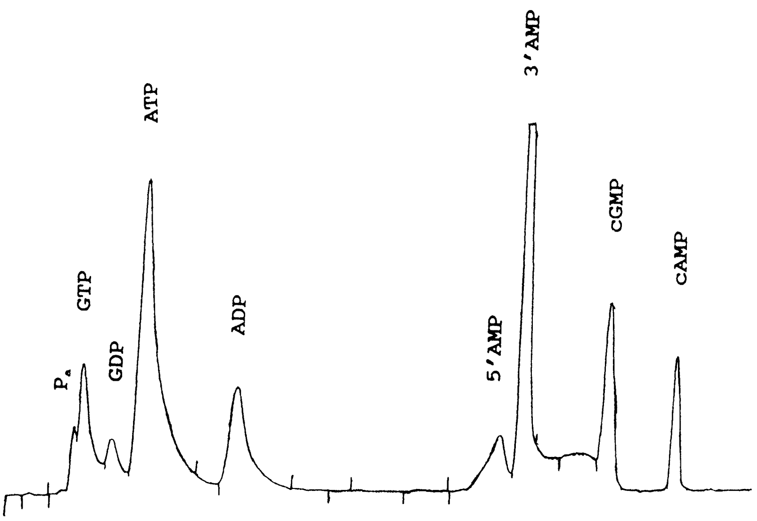

**Figure 1.** HPLC separation of nucleotide standards : GTP ( $t_R$ =1.40 min ), GDP ($t_R$ =1.96 min ), ATP ($t_R$=2.53 min), ADP ($t_R$=4.28 min), 5'AMP ($t_R$=9.21 min), 3'AMP ($t_R$=9.60 min), 3',5'-cGMP ($t_R$=11.21 min) and 3',5'-cAMP ($t_R$=12.52 min ).

The validation of the extraction technique was done by calculating the energy charge (EC) of the cells[12]:

$$EC = \frac{[ATP] + 1/2[ADP]}{[ATP] + [ADP] + [AMP]}$$

Most of the cells in a normal energy status have EC between 0.80 and 0.95[15]. For all our tissue samples EC oscillated between 0.80 and 0.92, indicating a good preparation of the extracts and, in the same time proving that lithium did not significantly modify the global energy status of the cells.

Fig.2 shows the separation of adenine- and guanine nucleotides from brain extracts. In controls (Fig.2.A.) the following nucleotides were identified: GTP co-eluted with GDP, ATP, ADP, 5'-AMP, 3'-AMP and 3',5'-cGMP. The signal with $t_R$= 12.33 min corresponding to 3',5'-cAMP in standard mixture was not present in this extract. Taking into account the fact that only basal, unstimulated AC activity was investigated and 3',5'-cAMP (whose levels are normally below $10^{-6}$ M) is metabolized within seconds to 5'-AMP[16,17], we considered that the effect of lithium on this second messenger could be appreciated only by the variation of 5'-AMP levels. $Li_2SO_4$ administration at increasing doses (from 2 to 10 mmol/kg) resulted in the disappearance of the peak with $t_R$= 9.92 min (corresponding to 3',5'-cGMP) and a decrease of the peak surface with $t_R$= 9.09 min, corresponding to 5'-AMP (Fig.2 B,C,D).

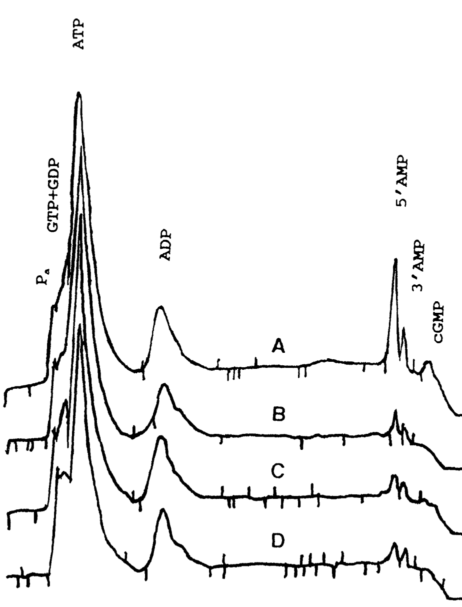

**Figure 2.** HPLC separation of nucleotides in brain homogenates, before (A) and after oral administration of 2 (B), 6 (C) and 10 (D) mmol Li/kg b.w.

The HPLC separation of adenine- and guanine nucleotides from kidney homogenates is depicted in Fig.3. From the controls' chromatograms were identified: GTP co-eluted with GDP, ATP, ADP, 5'-AMP, 3'-AMP and 3',5'-cGMP (Fig.3.A). In the chromatograms from $Li_2SO_4$-treated rats an increase of the surface of the peaks with $t_R$= 9.32 min (corresponding to 3'-AMP) and with $t_R$=9.93 minutes, (corresponding to 3',5'-cGMP) was observed (Fig.3 B,C,D).

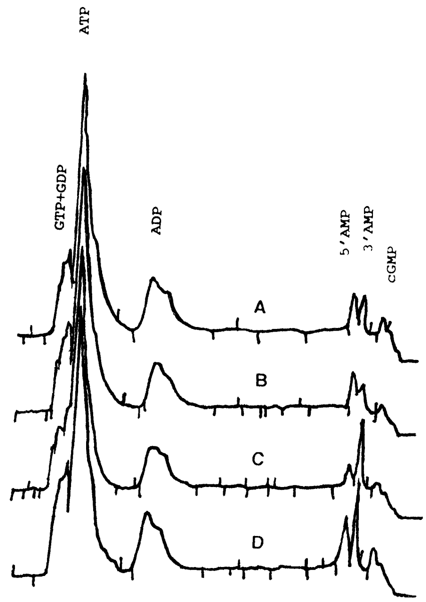

**Figure 3.** HPLC separation of nucleotides in kidney homogenates, before (A) and after oral administration of 2 (B), 6 (C) and 10 (D) mmol Li/kg b.w.

Fig.4 shows the separation of adenine- and guanine nucleotides from liver homogenates. In controls (Fig.4.A) the following nucleotides were identified : GTP co-eluted with GDP, ATP, ADP, 5'-AMP, 3'-AMP and a small peak corresponding to 3',5'-cGMP. In the chromatograms from $Li_2SO_4$-treated animals (Fig.4 B,C,D) the increase of the peak area corresponding to 3',5'-cGMP and a gradual decrease until disappearance of the peak with $t_R$=9.12 min, (corresponding to 5'-AMP) was observed.

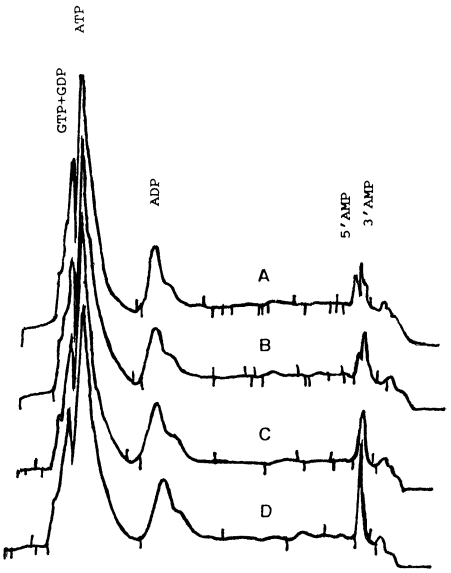

**Figure 4.** HPLC separation of nucleotides in liver homogenates , before (A) and after oral administration of 2 (B), 6 (C) and 10 (D) mmol Li/kg b.w..

The turnover of nucleotides in brain, kidney and liver is presented in Fig. 5, 6 and 7. The dose of 2 mmol/kg $Li^+$ induced the increase of ATP levels in brain (Fig.5), while the doses of 6 mmol/kg and 10 mmol/kg decreased in a smaller extent the levels of 5'-AMP. An inhibition of 3',5'-cGMP formation was observed for all $Li^+$ doses. No significant variations of nucleotides were observed in kidney (Fig 6) except the levels of 3',5'-cGMP, which were 4-5 times increased folowing all $Li^+$ doses. Fig.7 shows that $Li^+$, in all doses, in liver increased 5-6 times the 3',5'-cGMP levels while the doses of 6 and 10 mmol $Li^+$/kg inhibited the formation of 5'-AMP.

Unlike other studies, where the analysis of the cyclic nucleotides was performed by radioimmunoassay methods[18-20], our method allowed the concomitant separation and evaluation of both adenine- and guanine-dependant nucleotides in tissues. Although 3',5'-cAMP was not identified in these tissues, dose-dependent variations in the levels of its stable metabolite, 5'-AMP were observed. So, the acute administration of lithium induced a decrease of 5'-AMP levels in brain, which is more important when the dose of 2 mmol Li//kg ( extrapolated from the human therapeutic doses) was administered.

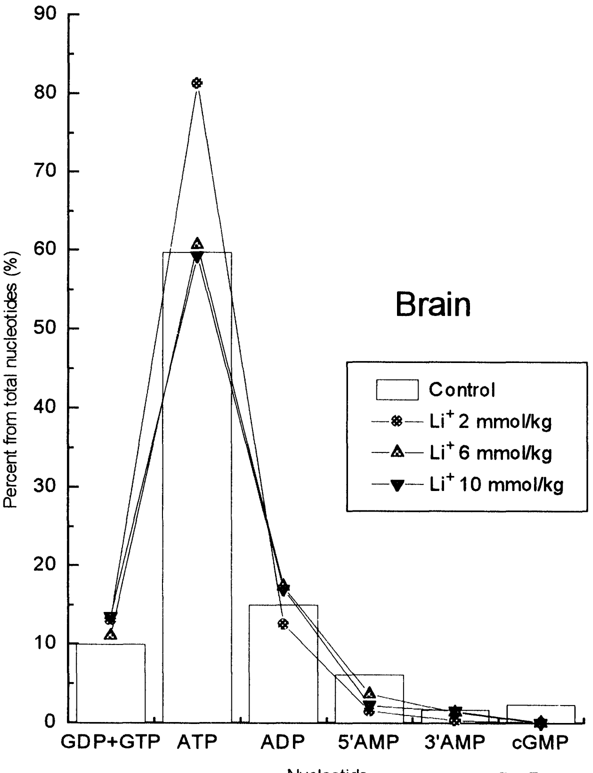

fig.5

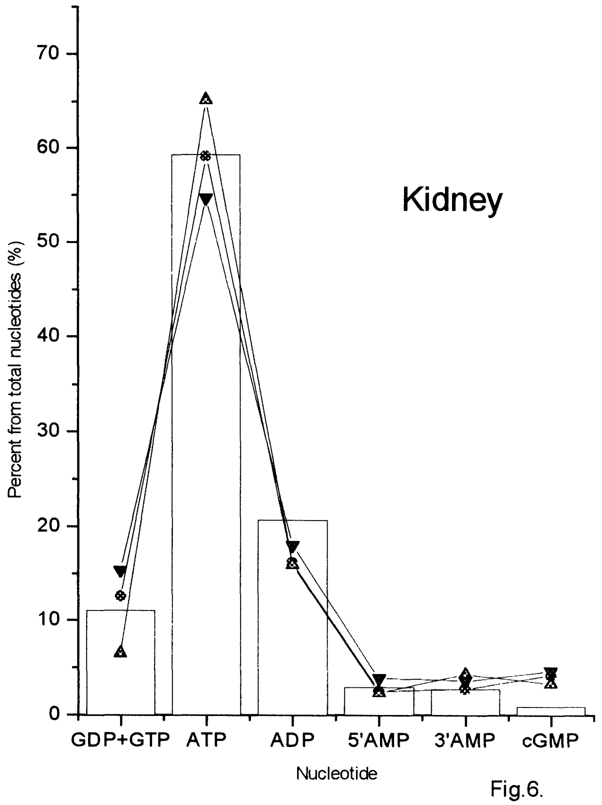

Fig.6.

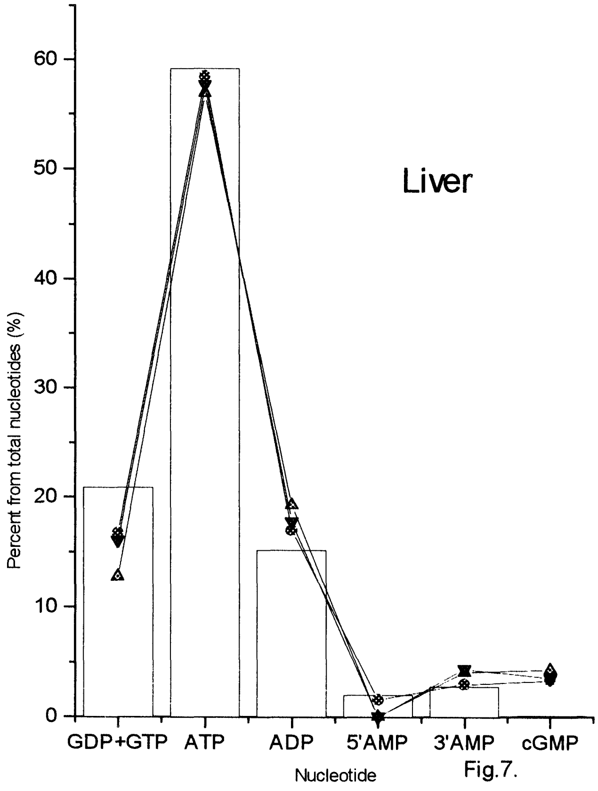

Fig.7.

**Figures 5-6-7.** Turnover of adenine- and guanine nucleotides in rat brain (5) , kidney (6) and liver (7) before (Control) and after oral administration of 2,6 and 10 mmol Li/kg b.w.

This might reflect an inhibition of the basal adenylate cyclase activity. In some studies the results were similar[11], while in others[19] the basal enzyme activity was reported to decrease only after chronic administration of lithium.

Unlike other results obtained in chronic experiments[11], where an antagonistic relationship between 3',5'-cAMP and 3',5'-cGMP after $Li^+$ treatment was reported, in our study all $Li^+$ doses inhibited the formation of 3',5'-cGMP in brain , concomitantly with the decrease of 3',5'-cAMP levels (Fig.5). These differences could be explained by the different type of administration (acute treatment) and assessement of cyclic nucleotides. In the same time, our results are in good agreement with the experiments of Kanba and Richelson[21] using cultured neuroblastoma cells, where the formation of 3',5'-cGMP was mediated by muscarinic receptors and inhibited in a dose-dependant fashion by increasing doses of LiCl above 1mM. These authors pointed out that some of lithium toxic effects resemble atropine poisoning and might be due to its antimuscarinic effects at the level of the receptor.

A significant increase of 3',5'-cGMP levels was observed in kidney, in all $Li^+$-treated groups while the 5'-AMP levels were not significantly affected by acute lithium administration (Fig.6).

The most important changes in the percentage of adenine- and guanine-dependent nucleotides were observed at the hepatic level, in a dose-dependant manner. Doses of 2 mmol $Li^+$/kg had a reduced effect on 3',5'-cAMP level, while the toxic doses of 6 and 10 mmol $Li^+$/kg inhibited the formation of 3',5'-cAMP (Fig.7). The explanation could be due to hepatocyte's specificity, with a high metabolic turnover, more susceptible comparatively to other organs.

Our study confirmed that the stimulation of 3',5'-cGMP formation by lithium might have important implications in its mechanism of toxicity. It is supposed that $Li^+$ may increase 3',5'-cGMP via modulation of the free fatty acids and their oxidized metabolites, especially prostaglandine $E_2$ and the associated free radicals which are known to activate guanylate cyclase[11].

Following acute administration, lithium's effects on 3',5'-cAMP were more significant at hepatic and brain level in a dose-dependant manner, while the effects on 3',5'-cGMP were different from brain to liver and kidney, respectively and were not dose-dependant. Thus, the relationships between 3',5'-cGMP and 3',5'-cAMP were dependant on the tissue investigated and in consequence, on the receptors and neurotransmiters involved.

Considering the different turnovers of 3',5'-cGMP and 3',5'-cAMP in different tissues, we suggest their use as relevant markers of lithium activity. Taking into account the most significant variations of 3',5'-cGMP in all investigated tissues, it might be prefered as a better indicator of lithium toxicity.

### REFERENCES

1. L.E.Hollister and J.C.Csemansky, *Clinical Pharmacology and Psychotherapeutic Drugs,* Churchill-Livingstone Inc., New York, 1990.
2. A.Georgotas and R.Cancro, *Depression and Mania,* Elsevier, New York, 1988.
3. M.P.V.Austin, F.G.Souza and G.M.Goodwin, *Br.J.Psychiatry,* 1991, **159**, 510.
4. A.Amdisen and J.Hildebrandt, *Psychother.Psychosom.,* 1988, **49**, 103.
5. A.J.Wood and G.M.Goodwin, *Psychological Medicine,* 1987, **17**, 579.
6. M.Chireux, *Médecine/Sciences,* 1994, **10**, 314.
7. A.Mork, A.Geisler and P.Hollund, *Pharmacol.Toxicol.*, 1992, **71**, 4.
8. P.P.Li, Y.-K.Tam, L.T.Young and J.J.Warsh, *Eur.J.Pharmacol.-Mol.Pharmacol.Sect.,* 1991, **206**, 165. 9. M.I.Masana, J.A.Bitran, J.K.Hsiao and W.Z.Potter, *J.Neurochem.,* 1992, **59**, 200.
10. G.Y.Sun, M.Navidi, F.-G.Yoa, T.-N.Lin, O.E.Orth, E.B.Stubbs and R.A.MacQuarrie, *J.Neurochem.,* 1992, **58**, 290.
11. B.Harvey, M.Carstens and J.Taljaard, *Neurochem.Res.,* 1993, **18**, 1095.
12. D.Perret, in *HPLC of Small Molecules, A Practical Approach,* (C.K.Lim Ed.), IRL Press, Oxford-Washington D.C., 1986, p.244.
13. D.L.Ramos and A.M.Schoffstall, *J.Chromatogr.,* 1983, **261**, 83.
14. F.S.Anderson and R.C.Murphy, *J.Chromatogr.,* 1976, **121**, 251.
15. L.Stryer, *Biochemie,* Flammarion Médecine-Sciences, Paris, 1985, p.266.
16. J.D.Rawn, *Biochemistry,* Neil Patterson Publishers, Burlington North Carolina, 1989, p.252.
17. E.Fournier, *Toxicologie. Biologie cellulaire appliquée à la sécurité des produits chimiques,* Ellipses, Paris, 1993.
18. E.D.Risby, J.K.Hsiao, H.K.Manji, J.Bitran, F.Moses, D.F.Zhou and W.Z.Potter, *Arch.Gen.Psychiatry,* 1991, **48**, 513.
19. H.R.Manji, J.A.Bitran, M.J.Masana, G.Chen, J.K.Hsiao, E.D.Risby, M.V.Rudorffer and W.Z.Potter, *Psychopharmacol.Bull.*, 1991, **27**, 199.
20. D.J.Sillence and C.P.Downes, *Biochem.Biophis.Acta,* 1992, **1138**, 46.
21. S.Kanba and E.Richelson, *New England J.Med.,* 1984, **310**, 1199.

**Received: January 31, 1996 - Accepted: April 17, 1996**

